# A diallel study to detect genetic background variation for FHB resistance in winter wheat

**DOI:** 10.1038/s41598-024-53710-z

**Published:** 2024-02-26

**Authors:** Bipin Neupane, Bradley Bisek, Francois Marais

**Affiliations:** 1 Department of Plant Sciences, North Dakota State University, Fargo, ND 58108 USA; 2 Department of Plant Pathology, Washington State University, Pullman, WA 99164 USA

**Keywords:** Genetics, Plant sciences

## Abstract

Breeding for resistance to Fusarium head blight (FHB) relies strongly on a limited number of larger-effect resistance QTL that have been mapped and associated with nearby markers. Smaller-effect (background) resistance QTL may also contribute moderate levels of resistance yet are mostly poorly characterized. Overall resistance of a genotype is determined by the combined action of both types of resistance QTL. This study aimed to identify well-adapted, advanced hard red winter (HRW) wheat breeding lines with useful background resistance QTL. A diallel trial consisting of 11 parents and 55 non-reciprocal F_1_ hybrids was tested for Type II FHB resistance in a replicated greenhouse experiment. Significant differences were detected among entries for disease severity (DS), general combining ability (GCA) and specific combining ability (SCA) with four parents being identified as the best general combiners with lowest DS. The ratio of GCA:SCA effects suggested that additive QTL effects were of primary importance. Overall, resistance QTL showed incomplete dominance, an excess of dominant alleles, and a greater contribution of positive effect genes. F_2_ of the six best F_1_ hybrids with the lowest DS were compared in a second greenhouse FHB trial to select possible transgressive segregates for continued evaluation and line development.

## Introduction

Wheat is a highly valued crop that ranks second in terms of global cereal production, with around 781.31 million tons produced in 2022/23^1^. Hexaploid wheat (*Triticum aestivum* L., 2n = 6x = 42, AABBDD) and durum wheat (*Triticum turgidum*, L., 2n = 4x = 28, AABB) account, respectively, for 95% and 5% of wheat production worldwide^2^. Wheat is a primary ingredient of a wide variety of foods products including bread, cereals, pastries, pasta, crackers, cookies, etc. that are commonly consumed^3^. The world's leading wheat producers are Russia, China, India, the United States, Canada, and the European Union^4^. To provide for current population growth and manage the risk of increasing global hunger, wheat production will need to be doubled by 2050^5^; however, wheat production is hindered by a variety of biotic and abiotic stresses^6^.

Fusarium head blight (also known as scab) is a major fungal disease of small cereal grains such as wheat, barley, and oats^7^. The disease is caused by different species of the *Fusarium* genus, but *Fusarium graminearum* Schwabe is the most damaging^7,8^. Throughout the twentieth century, severe FHB epidemics were sporadically experienced in Europe, Asia, and North and South America^9^. Since its reemergence in North America in the 1990s, FHB epidemics of varying intensity have been recurring frequently^10^, reducing grain yield, grade, and end-use quality; limiting crop rotation options; limiting variety choice and requiring expensive control measures^7^. Mycotoxins that remain in infected grain pose significant food safety risks and greatly reduce its market value^11^.

Commonly used management measures to control FHB are host resistance, fungicide application, biological control, and cultural practices, each of which is only partially effective^12^. Genetic resistance to FHB is determined by multiple small-effect quantitative trait loci (QTL) with complex inheritance, and their expression is modified by genotype and environment (GXE) interaction^13^. Five types of active FHB resistance mechanisms have been reported, including Type I (to pathogen infection^14^), Type II (spread within the spike^14^), Type III (kernel infection^15^), Type IV (tolerance^15^) and Type V (toxin resistance, either by degrading pathogen-produced toxins or through plant insensitivity to toxin accumulation^16^). Type I and Type II resistance are the two main types of resistance mechanisms against FHB in wheat^14^, with Type II resistance being more widely utilized due to its broader-spectrum protection and the presence of multiple major and minor genes that make it more effective^17^. In contrast, Type I resistance has specificity and narrow-spectrum protection, limiting its effectiveness in controlling different strains of the FHB pathogen^15^.

The North Dakota State University HRW wheat breeding program conducts pre-breeding to introgress well-characterized, effective FHB resistance QTL from external sources. Among these, *Fhb1* (chromosome arm 3BS) was reported to provide stable type II resistance^18–20^. *Qfhs.ifa-5A* is a strong-effect FHB resistance QTL on chromosome arm 5AS that provides type I resistance^21^, and to a lesser extent, also confers resistance of type II^22^. Two significant resistance QTL discovered on chromosome 5A in the wheat accession PI 277012 were named *Qfhb.rwg-5A.1* (5AS) and *Qfhb.rwg-5A.2* (5AL), respectively^23^. Both latter QTL were reported to contribute type I and type II resistance, as well as resistance to DON accumulation^23^. During introgression of the four resistance QTL, it became apparent that their expression in winter wheat genetic backgrounds was highly variable and strongly dependent on the presence or absence of unidentified “background” or “native” resistance QTL^24^. Background FHB resistance has been well documented and, in the US, well-known cultivars like Wesley, Lyman, Overland, Ernie, and Freedom are recognized sources of effective native resistance against FHB^25^.

Diallel cross analysis is frequently used in genetic research to study the inheritance of a trait within a chosen set of genotypes and to identify superior parents for hybrid production or cultivar development^26,27^. The method allows for calculation of the relative importance of additive and non-additive gene action in the expression of a trait, provides comprehensive assessment of combining ability and breeding potential of the parents, and unveils the probable mode of gene action^28–30^. In the present study, diallel analysis was done to 1—evaluate 11 elite HRW wheat parents for Type II background FHB resistance and combining ability; 2—study the mode of gene action; and 3—identify the best parent(s) for use in future breeding program crosses aimed at the introgression of exotic FHB resistance QTL. Following the F_1_ analysis, an attempt was also made to select potential transgressive segregates with increased FHB resistance from the F_2_ of the best crosses and to initiate single seed descent (SSD) inbreeding for ongoing selection. This study primarily assessed Type II resistance to FHB and the evaluation of Type I resistance was not included. Furthermore, resistance testing was done under controlled greenhouse conditions rather than during a natural field epidemic, meaning that conclusions based on the trial results should be verified in future field trials.

## Results and discussion

### Evaluation of the F_1_ (greenhouse FHB Trial 1)

The disease severity (DS) data (percentage) were not normally distributed; however, the data were not transformed before analysis of variance (ANOVA) as the sample sizes were deemed large enough for the central limit theorem^31^ to apply. ANOVA of the DS data was conducted which indicated a significant difference among the parents and the 55 hybrids tested in the greenhouse (Table 1).

**Table 1:** ANOVA of FHB Type II disease severity measured in an 11 × 11 half diallel greenhouse trial. ** indicates that a value differs significantly from zero at the 1% level.

	DF	Sum Squares	Mean Squares	F value	Pr (> F)
Entries	65	62063	954.82	9.2830	< 2.2e−16 **
Replications	3	483	160.92	1.5645	0.1985
Entries × Replications	195	27371	140.36	1.3646	0.0102*
Residuals	252	25920	102.86		

The DS averages of the 66 entries are summarized in Table 2. With regard to the parents, the values ranged from 13.2% to 55.4%, whereas the F_1_ ranged from 6.5% to 53.7%. Five parents (18Nord-107, 19Nord-122, 19Nord-131, 19Nord-129, and ND Noreen) exhibited higher FHB resistance, with average DS ranging from 13.2% to 23.7%. Of these five parents, 19Nord-131 tested positive (markers) for the presence of FHB resistance QTL, *Fhb1* and *Qfhs.ifa-5A*. The ten possible F_1_ combinations among the five most resistant parents had mean DS values ranging from 6.5% to 22.9%. The average DS values of the parental arrays of the top five parents (each based on four F_1_) ranged from DS = 13.0% to DS = 18.1%. Two F_1_ combinations (18Nord-107/19Nord-129 and 18Nord-107/ND Noreen) from the more resistant parent group had the lowest average DS values in the trial, with DS 6.51% and 6.98%, respectively.

**Table 2:** Average (observed) disease severity of the parents (diagonals) and F_1_ combinations (off-diagonals) as measured in the greenhouse.

Parent number and name	1	2	3	4	5	6	7	8	9	10	11	
	17Nord-96	18Nord-103	18Nord-107	19Nord-122	19Nord-131	19Nord-129	ND Noreen	20SenA-33	20Nord-136	20JuniorH-36	20JuniorP-17	Array average
1. 17Nord-96	40.2	17.79	23.69	27.34	20.43	20.58	13.16	42.90	30.70	37.79	34.70	26.91
2. 18Nord-103		30.84	18.21	24.09	17.96	30.38	13.87	45.33	21.40	30.37	20.71	24.01
3. 18Nord-107			**23.65**	**19.89**	**22.87**	**6.98**	**6.51**	23.20	15.35	21.80	20.72	17.92
4. 19Nord-122				**17.02**	**16.04**	**16.39**	**19.92**	38.82	21.43	27.17	41.25	25.24
5. 19Nord-131					**16.74**	**15.32**	**16.52**	38.04	29.91	28.65	25.42	23.12
6. 19Nord-129						**15.73**	**13.17**	39.34	22.17	38.89	32.10	23.53
7. ND Noreen							**13.17**	21.40	13.61	30.30	25.43	17.39
8. 20SenA-33								55.37	45.09	53.69	40.83	38.86
9. 20Nord-136									31.31	37.04	29.80	26.65
10. 20JuniorH-36										32.05	40.83	34.65
11. 20JuniorP-17											44.65	31.18

The average value of the ten progeny associated with a parent is given in the last column. The five most resistant parents and the ten F_1_ combinations among them are given in bold.

Among the 11 parents, 20SenA-33, 20JuniorP-17, and 17Nord-96 were the most susceptible to FHB, with average DS ranging from 40.2% to 55.4% (Table 2). It is noteworthy that parent 20JuniorP-17, despite having the *Fhb1* resistance gene marker present, displayed the second-highest susceptibility (DS = 44.7%) in the group. This confirms that the presence of *Fhb1* does not always guarantee strong Type II FHB resistance, as susceptibility or modifier/inhibitor QTL in the genetic background may mask its effect^32^. Since only two parents are known to express DNA marker polymorphisms consistent with the presence of mapped resistance QTL (20Junior-P17 for *Fhb1* and 19Nord-131 for *Fhb1* and *Qfhb.ifa-5A*), it is highly likely that there are genetic differences in background resistance among the 11 winter wheat parents (Table 2).

### Estimation of general and specific combining ability

Table 3 summarizes the results of the analysis of variance of the DS data following Griffing’s^28^ method which provides a statistical analysis of main effects (GCA) and interactions (SCA). There was significant variation for both GCA and SCA with respect to FHB Type II resistance, indicating strong contributions of both additive and non-additive gene effects in the hybrid combinations. The mean squares of GCA (MS_GCA_) and SCA (MS_SCA_) were 602.60 and 34.03, respectively (Table 3). The GCA/SCA combining ability ratio was close to unity (0.97), which suggested that additive genetic components played a bigger role in determining FHB resistance in the lines studied^33^. A pure line selection strategy such as the Pedigree breeding method relies on the presence of adequate additive genetic variation and can be used for the improvement of a targeted trait. Thus, identifying parents with high GCA that contribute to additive genetic effects is crucial when planning crosses to breed more resistant cultivars. Significant SCA in the F_1_ generation indicated that in addition to the strong additive effects, non-additive genetic effects were also important. As inbreeding progresses in subsequent segregating generations, the relative contribution of non-additive effects to differences among lines will decrease.

**Table 3:** ANOVA table showing the significance of GCA and SCA effects.

	DF	Sum squares	Mean Squares	F value	Pr (> F)
GCA	10	6026.0	602.60	32.6864	< 2.2e-16**
SCA	55	1871.9	34.03	1.8461	0.001239**
Error	195	3595.0	18.44		

#### GCA-effects

The GCA effects of the parents for disease severity ranged from − 9.32 to 13.68 (Table 4). Negative GCA values suggested increased resistance, while positive values indicated increased susceptibility. Of the eleven parental lines, eight showed significant negative GCA effects, but only five of the effects increased resistance (Table 4). The latter five parents (18Nord-107, 19Nord-122, 19Nord-131, 19Nord-129, and ND Noreen) had the best actual DS scores (Table 2) as well as strongest GCA effects. ND Noreen (GCA-effect = − 9.32; DS = 13.17%) was the most resistant of the parents and was also the best general combiner. The 19Nord-131 resistance and GCA effect may have been largely due to the likely presence of *Fhb1* and *Qfhb.ifa-5A*, and it was therefore not considered a potential source of background resistance; however, the remaining four parents may possess unknown resistance QTL that could contribute significant background resistance. F_1_ combinations of the top three general combiners 18Nord-107/ND Noreen (DS = 6.5%), 18Nord-107/19Nord-129 (DS = 7.0%), and 19Nord-129/ND Noreen (DS = 13.2%), had the lowest DS scores and ranked first, second, and third among the 66 trial entries (with five entries in third place). The presence of parents with such strong GCA effects suggested a strong likelihood of developing inbred lines with resistance that equals or surpasses that of the parents.

**Table 4:** Calculated GCA (diagonal values) and SCA (off-diagonal values) effects.

Parent number and name	1	2	3	4	5	6	7	8	9	10	11
	17Nord-96	18Nord-103	18Nord-107	19Nord-122	19Nord-131	19Nord-129	ND Noreen	20SenA-33	20Nord-136	20JuniorH-36	20JuniorP-17
17Nord-96	2.16	− 9.65**	2.03	1.08	− 4.15	− 4.16	− 6.46	0.26	1.15	1.97	− 0.37
18Nord-103		− 1.51	0.24	1.51	− 2.94	9.31*	− 2.07	6.37	− 4.45	− 1.76	− 10.6**
18Nord-107			− 7.30**	**3.10**	**7.75**	− 8.30*	− **3.64**	− 9.97**	− 4.72	− 4.53	− 4.88
19Nord-122				− 2.69*	− **3.67**	− **3.49**	**5.15**	1.04	− 3.24	− 3.77	11.03**
19Nord-131					− 4.37**	− **2.88**	**3.42**	1.94	6.90	− 0.62	− 3.12
19Nord-129						− 4.20**	− **0.08**	3.06	− 0.99	9.44*	3.39
ND Noreen							− 9.32**	− 9.75**	− 4.43	5.97	1.84
20SenA-33								13.68**	4.02	6.36	− 5.77
20Nord-136									0.58	2.80	− 3.70
20JuniorH-36										6.85**	1.05
20JuniorP-17											6.12**

*, ** indicates that a value differs significantly from zero at the 5% and 1% levels, respectively.

The five parents that had the most resistant phenotypes and the ten F_1_ combinations among them are given in bold.

#### SCA-effects

The SCA effects for disease severity ranged from − 10.68 to 11.03 (Table 4). Among the 55 SCA effects, eight were significant, with five increasing and three decreasing resistance. Among the five significant SCA effects that increased FHB resistance, F_1_ 18Nord-107/19Nord-129 resulted from a cross between two of the five best general combiners (which also had low DS scores). Therefore, the latter F_1_ likely had a strong additive component to its SCA effect. The SCA effects in crosses ND Noreen/20SenA-33 and 18Nord-107/20SenA-33 each involved a high GCA parent and was likely due to both additive and non-additive gene effects, as one parent had good resistance and significant GCA while the other parent was highly susceptible with low GCA. The two remaining crosses, 18Nord-103/17Nord-96 and 18Nord-103/20JunH-36, had moderately susceptible or susceptible parents with poor GCA, and the SCA effect could have had a strong non-additive component. For example, the F_1_ 17Nord-96/18Nord-103 exhibited stronger resistance than its parents, probably due to a strong contribution from non-additive gene effects (SCA-effect = − 9.65; observed DS = 17.8%, while parents had DS of 40.3% and 30.8%, respectively).

### Hayman analysis

Griffing’s^28^ analysis was followed by Hayman’s^29^ for an assessment of the genetic architecture of FHB resistance. Table 5 presents the results of an analysis of variance that allows for estimation of variance components that can help to elucidate the genetic mechanisms causing variation in type II FHB resistance in the data set. The analysis confirmed the presence of significant additive genetic variation (a) and non-additive genetic variation (b) (Table 5). The estimates of b_2_ (gene asymmetry) and b_3_ (non-additive deviations unique to each F_1_) were also significant. The additive genetic variance was found to be larger than the non-additive genetic variance, indicating its greater importance. The uniformity test (t^2^ test) results were also non-significant for DS, supporting the validity of Hayman's diallel analysis assumptions and suggesting the absence of significant epistatic (excluding additive X additive) interactions among the genes responsible for DS^29^.

**Table 5:** Results obtained following an analysis of variance (Hayman^29^) of FHB disease severity data from the F_1_ diallel experiment.

	DF	Sum squares	Mean Squares	F value	Pr(> F)
Blocks	3	264.99	88.33	1.19	0.31
Entry	65	31591.42	486.02	6.59	5.99e−25**
Additivity (a)	10	10300.81	1030.08	13.96	0.00 **
Non-additivity (b)	55	21289.62	387.08	5.24	0.00**
b_1_	1	5.2315	5.2315	0.07	0.79
b_2_	10	2978.98	297.89	4.03	0.00**
b_3_	44	18305.40	416.03	5.64	0.00**
Total × Blocks	195	14379.84	73.74		
a × Blocks	30	2645.44	88.18		0.31
b × Blocks	165	11734.24	71.11		0.55
b_1_ × Blocks	3	32.27	10.75		0.41
b_2_ × Blocks	30	2367.52	78.91		0.54
b_3_ × Blocks	132	9334.43	70.71		0.53
Residuals	195	14379.84	73.742		

(a) = additive genetic variance; (b) = non-additive genetic variance; b_1_ = tests the overall difference between parental and F_1_ means (direction of dominance), b_2_ = measures consistency of mean dominance deviation over arrays (gene asymmetry), and b_3_ = measures non-additive deviations unique to each F_1_.

The variance components and genetic parameters were calculated and utilized by the software to calculate informative genetic ratios. The narrow sense heritability of the trait was estimated to be 0.71, while the broad sense heritability was 0.86. These values confirmed a strong influence of additive genetic effects and suggest that selection for the trait is likely to result in significant genetic gain. The average degree of dominance for disease severity was 0.84 (Table 6), indicating incomplete dominance as values less than 1 would signify.

**Table 6:** Variance components and genetic parameter estimates for FHB disease severity in a diallel experiment that was analyzed according to Hayman^29^.

	Estimates
Parent mean	29.15
Variance of parents	183.37
Mean variance of arrays	77.86
Variance of the means of arrays	48.44
Mean covariance between parents and arrays	81.29
E (expected environmental component of variance)	18.49
D (additive genetic variance)	164.88
F = Mean of Fr over the arrays, where Fr is the covariance of additive and dominance effects in single arrays)	11.31
H1 (dominance variance)	117.55
H2 = H1[(1 − (U − V)2)] where U and V are the proportions of positive and negative genes in the parents	80.70
h2 (dominance effect)	20.61
^1^Average degree of dominance (= H1/D)1/2	0.84
^3^Proportion of genes with positive or negative effects in the parents	0.17
^2^Ratio of dominant and recessive genes in the parents	1.08
r between Wr + Vr and Yr	0.33
Prediction for measure of comp. dominant & recessive parent	0.11
Additive variance	54.81
Phenotypic variance	128.56
Genotypic variance	54.81
Mean covariance of additive and dominance effects	11.31
Variance for components of variation	150.86

^1^0 = No dominance; > 0 > 1 = Partial dominance; 1 = Complete dominance; > 1 = Over-dominance.

^2^1 = Equal proportions; < 1 = Excess of recessive genes; > 1 = Excess of dominant genes.

^3^Equally divided = 0.25.

**Figure 1:**
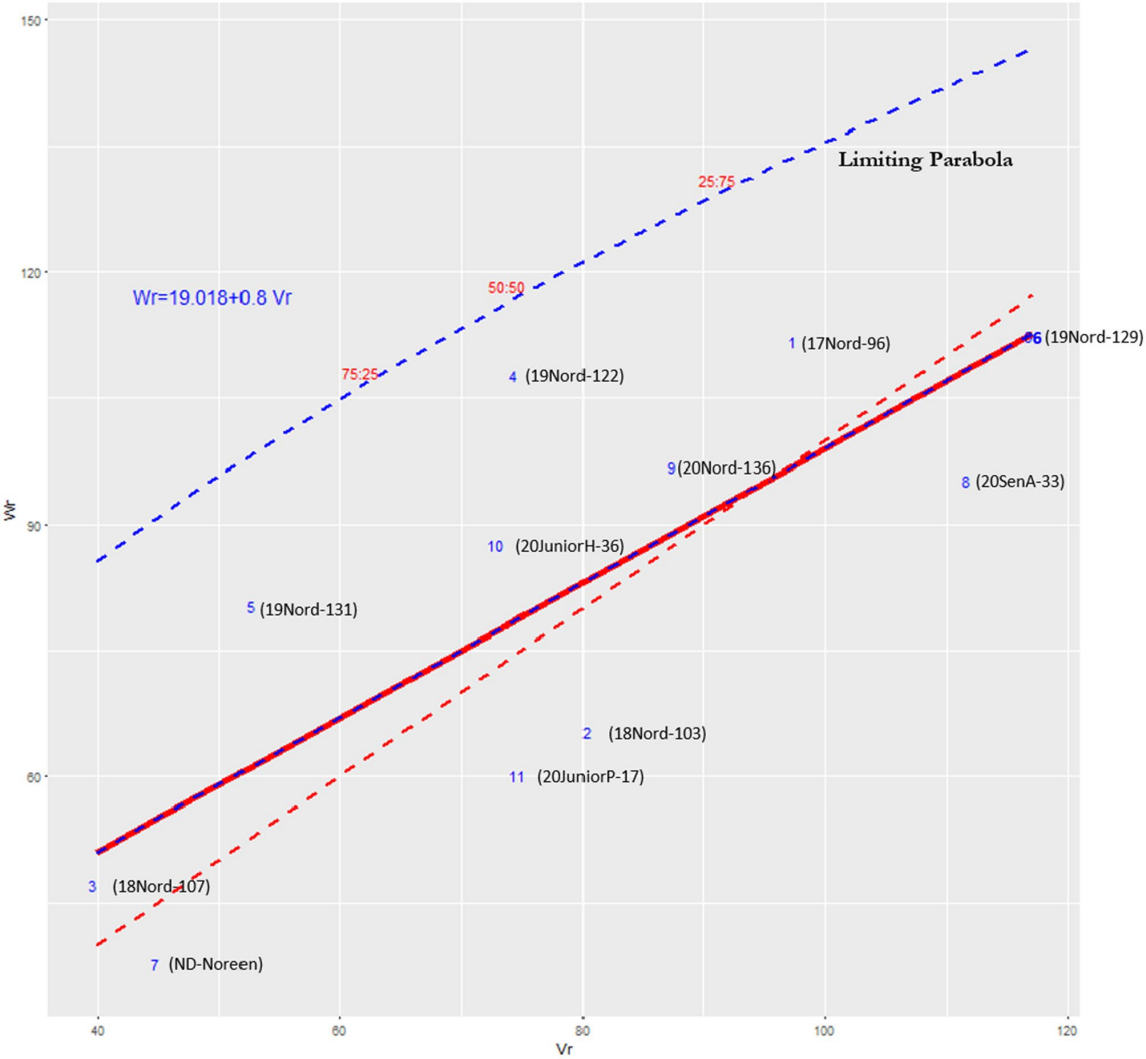
Wr–Vr graph where Vr represents the variance of arrays and Wr represents the covariance between parents and offspring.

The Wr–Vr graph^29^ is shown in Fig. 1. The slope of the regression line (Wr = 19.018 + 0.8Vr) is close to, and not significantly different from unity, suggesting that the observed DS scores resulted primarily from allelic (additive and dominance) gene action rather than non-allelic/epistatic gene action. Overall, the resistance QTL showed incomplete/partial dominance as the regression line intercepts the Wr axis above the point of origin. The calculated proportion of dominant to recessive genes affecting Type II resistance in the 11 parents was 1.08 (Table 6), suggesting an excess of dominant genes. The proportion of genes with positive and negative effects in the parents was 0.17 (Table 6) which deviated from 0.25 (which indicates symmetry), suggesting an excess of genes that reduced DS (improved FHB resistance). On the Wr-Vr graph, the order of dominance with respect to each parent is reflected by its position on the regression line: parents that carry mostly dominant genes are positioned closer to the origin of the regression line, while parents carrying mostly recessive genes are positioned further away. Parents with equal or close to equal proportions of dominant and recessive genes occur in the middle of the range. The direction of dominance (increased or decreased DS) was revealed using two criteria^34^: First, the positive correlation (r = 0.33) between the parental means for DS and the value of Wr + Vr (Table 6) suggested that dominance tended to decrease DS (increased resistance). Second, parents with the lowest DS (Table 2) coupled with the lowest Wr + Vr value (18Nord-107, ND Noreen in Fig. 1) contributed dominant QTL that tended to increase resistance (reduce DS). On the contrary, 17Nord-96 had high DS and a high Wr + Vr value, suggesting the presence of recessive genes that increased susceptibility. 19Nord-129 had low DS coupled with a high Wr + Vr value suggesting the presence of recessive genes that enhanced resistance.

Focusing on the five parents that had the best average DS scores and exhibited the strongest (and highly significant) GCA effects (Tables 2 and 3), the following conclusions could be drawn:

- Parents 18Nord-107 (GCA effect − 4.37**), ND Noreen (GCA-effect − 9.32**), and 19Nord-131 (GCA effect − 4.37**) were located closer to the origin on the Vr-Wr graph, suggesting that the three lines possess mostly dominant genes with increasing effect on resistance/susceptibility. This result was anticipated for 19Nord-131 since it is believed to have both *Fhb1* and *Qfhs.ifa-5A*. Parents 18Nord-107 (pedigree = AC Emerson/Ideal) and ND Noreen (pedigree = Decade/Armour) do not carry any well-characterized, known larger effect resistance QTL and therefore exhibit native genetic background resistance that is primarily due to dominant QTL. The latter two parents are not closely related and may have genetically distinct sets of resistance QTL. The Canadian cultivar AC Emerson, included in the pedigree of 18Nord-107, is known to have intermediate FHB resistance derived from the cross between McClintock/CDC Osprey^35^. Similarly, ND Noreen is known to possess genetic background resistance^24^ that is comparable to the level of resistance in AC Emerson. The parents of ND Noreen, Decade (released in 2010 by Montana State University, Supplementary Information 1) and Armour (released by Westbred in 2008, Supplementary Information 2), do not include introgressed, larger effect QTL.
- Parent 19Nord-129 (GCA effect − 4.20**) was located furthest from the Wr-Vr origin, indicating that it carries mostly recessive genes for resistance/susceptibility. Since 19Nord-129 (pedigree = Klatt-19//Falcon/RCUOGDHACF110902D) exhibits very good general combining ability it may represent a third and distinct source of background resistance. In its pedigree, RCUOGDHACF110902D is known to possess significant FHB resistance and was provided in 2011 by Dr L. Tamburic-Ilincic; Univ Guelph, Ontario, Canada. Although the origin of the line differs from the US material, the pedigree and whether it possesses known "larger effect" QTL genes remain unknown. Nevertheless, it can still be a valuable parent and is believed not to possess *Fhb1*.
•19Nord-122 (GCA effect = 2.69*) was situated at an intermediate position on the Wr-Vr graph, indicating contributions from both dominant and recessive genes.
•Based on the above results, parents ND Noreen, 18Nord-107 and 19Nord-129 were the most promising sources of FHB Type II genetic background resistance. The three genotypes appeared to contribute different sets of background resistance QTL which presented the opportunity to breed for even stronger background resistance. The F_2_ of their intercrosses were therefore included in the F_2_ FHB trial to initiate pure line selection for Type II resistance.
•Crosses of ND Noreen, 18Nord-107 and 19Nord-129 with 19Nord-131 presented an opportunity to pyramid background resistance with *Fhb1* and *Qfhs.ifa-5A*. The three crosses were therefore also included in the F_2_ FHB trial.

### Screening of specific F_2_ families for Type II resistance in Greenhouse FHB trial 2

The F_2_ of six cross combinations along with their parents and controls, 19Nord-122 and 20SenA-33, were evaluated for Type II FHB resistance in another greenhouse trial. An ANOVA was done using the replicated DS data of the parents and controls (Table 7). There were highly significant differences among the entries. The average DS of the parents and controls in the F_2_ trial ranged from 13.61% to 32.31% (Table 8) and correlated well (r = 0.86*) with the corresponding means from the F_1_ trial. Table 8 also shows the F_2_ population averages of the six selected crosses. These average DS values fell within the range of 13.62% to 23.23%, and the correlation coefficient between the means of the F_1_ and F_2_ was r = 0.64^ns^. This lower correlation was anticipated due to the narrow range of DS within which the (selected for resistance) populations occurred. Thus, the two FHB trials appeared to rank the tested genotypes fairly consistently.

**Table 7:** ANOVA results for FHB Type II disease severity measured in four parents and two controls in a second greenhouse trial.

	DF	Sum squares	Mean squares	F value	Pr (> F)
Genotypes	5	8502.1	1700.43	19.806	< 2.2e−16**
Residuals	239	20518.9	85.85

**Table 8:** Average F_2_ disease severity of four parents, two controls and six cross combinations that were evaluated in the second greenhouse trial. Corresponding averages from the F_1_ trial were included for ease of comparison.

Genotype	Resistance type^1^	Genotypic mean DS		Cross	Resistance type^1^	Population mean DS	
		F_1_	F_2_^2^			F_1_	F_2_
ND Noreen	B	13.17	17.42^c^	18Nord-107/19Nord-129	B × B	6.98	18.93
18NORD-107	B	23.65	21.93^b^	ND Noreen/18Nord-107	B × B	6.51	13.62
19Nord-129	B	15.73	13.61^c^	ND Noreen/19Nord-129	B × B	13.17	22.8
19Nord-131	I	16.74	21.39^b^	ND Noreen/19Nord-131	B × I	16.52	16.91
19Nord-122	C	17.02	17.27^c^	18Nord-107/19Nord-131	B × I	22.87	23.23
20SenA-33	C	55.37	32.31^a^	19Nord-129/19Nord-131	B × I	15.32	19.86

^1^B = Background resistance, I = Introgressed resistance (*Fhb1*; *Qfhb.ifa-5A*), C = control.

^2^Significance of differences among genotypic means (95% confidence interval) are indicated.

### Selection of plants with the most resistant phenotypes in six F_2_ populations

**Figure 2:**
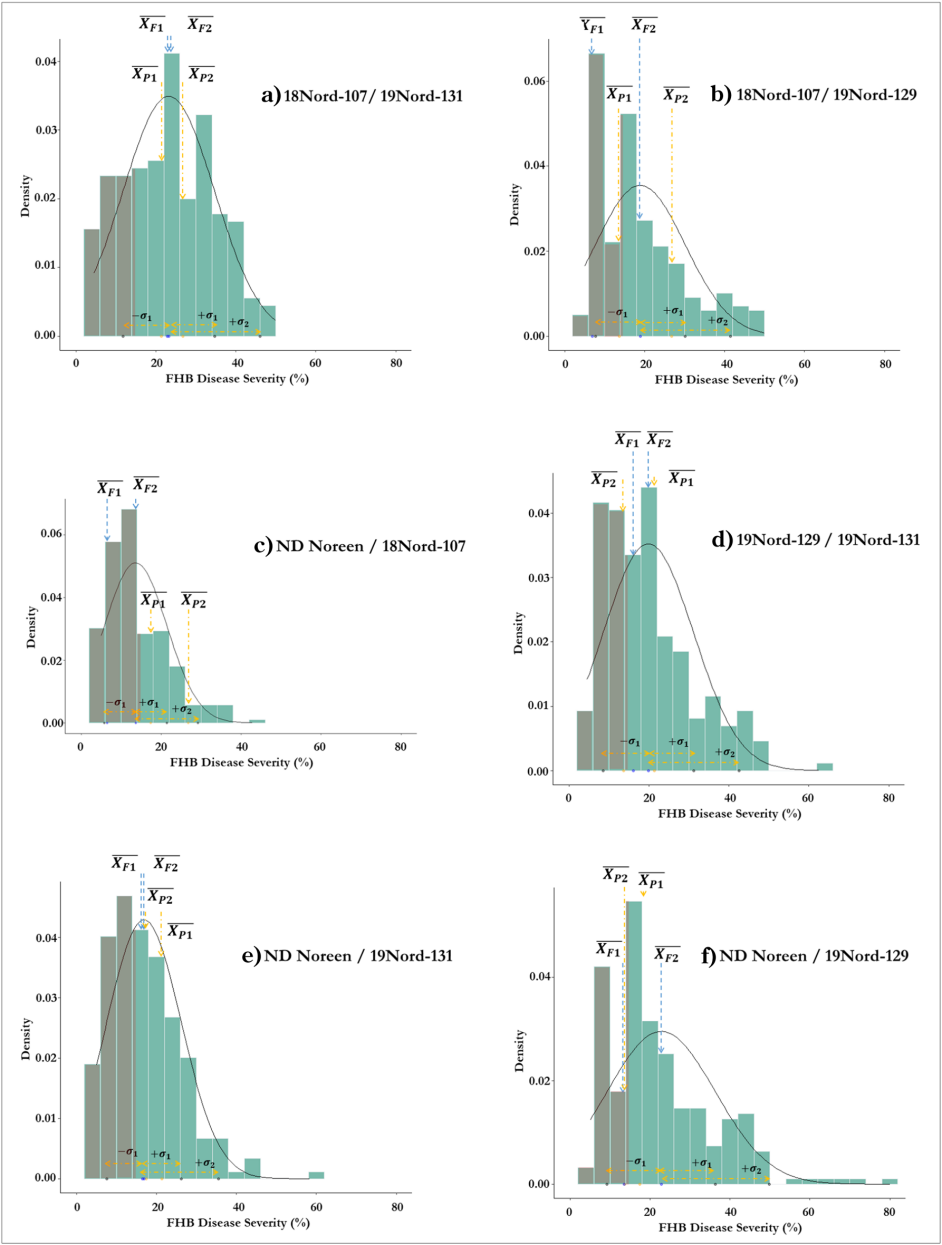
Histogram and a normal distribution curve fitted on the disease severity data of each of six crosses where each figure (a-f) shows the relative positions of the parental means (($\overline{{X }_{P1}}$) & ($\overline{{X }_{P2}}$)) and the mean performance of that cross in the first $(\overline{{X }_{F1}}$) and second trials ($\overline{{X }_{F2}}$). Gray color indicates the number of plants selected based on 14% arbitrary disease severity.

To identify possible transgressive segregates with superior FHB resistance, the most resistant plants from the six F_2_ populations were chosen for further inbreeding and testing. To determine a cut-off for retaining the more resistant segregates, the data of the four parental lines were first analyzed. For each parent, the lowest, mean, and highest DS values were considered. An arbitrary threshold DS value of 14% was chosen as the selection criterion, as this would retain a manageable number of F_2_ selections while ensuring that the most resistant plants were not excluded. Histograms that depict the distribution of mean DS of single plants within each of the six F_2_ populations are shown in Fig. 2. An approximating normal curve, relevant parental, F_1_ and F_2_ population means, and selection detail are also provided (Fig. 2).

Within those crosses that will continue to be selected for improvement of background resistance, 18Nord-107/ND Noreen had the lowest mean DS (13.62%) and produced the highest number of selections (39 plants with DS averages lower than 14%). Crosses 18Nord-107/19Nord-29 (DS = 18.93) and ND Noreen/19Nord-129 (DS = 22.8) yielded 19 and 10 selections, respectively. A total of 68 plants were retained from this group.

With regard to the crosses with 19Nord-131 (*Fhb1* and *Qfhs.ifa-5A*): From cross ND Noreen/19Nord-131 (mean F_2_ DS = 16.91), 26 plants had DS lower than 14%. In cross 18Nord-107/19Nord-131 (mean F_2_ DS = 23.23), 11 plants were selected and from cross 19Nord-129/19Nord-131 (mean F_2_ DS = 19.86), 18 plants were selected. A total of 55 plants were kept. The DS values of the 94 segregates that were chosen were lower than the ND Noreen average (DS = 17.4%), suggesting that the group could include highly resistant plants. The F_3_ will be grown (greenhouse) for multiplication and scrutiny for phenotype, including robustness, fertility, and plant height. From this selection, the F_4_ generation will be field tested and head selection will be done to establish F_5_ inbred lines. This material has significant potential as a valuable resource that can produce superior breeding parents and useful inbred lines for the continued improvement of FHB resistance.

## Conclusion

The 11 parents differed significantly for FHB Type II resistance. Additive genetic effects were predominant, indicating that dedicated pure line selection is likely to be effective for improving overall resistance. The narrow sense heritability was estimated to be 0.71, which would predict modest to good expected genetic gain from selection. Based on the analyses proposed by Griffing^28^ and Hayman^29^, three parents with the lowest DS and highest GCA were 18Nord-107, ND Noreen, and 19Nord-129. It appeared that these parents possess useful and diverse background resistance that may involve various FHB resistance QTL. An attempt will be made to select for better background resistance within segregating progeny of the three best lines. The three best parents also constitute valuable genetic backgrounds for pyramiding with known, larger-effect resistance QTL to achieve higher levels of FHB resistance. In addition to the three F_1_ combinations that were obtained from among the three best parents, their hybrids with 19Nord-131 (believed to have *Fhb1* plus *Qfhs.ifa-5A*) were also included in an F_2_ greenhouse trial to select possible transgressive segregates. The progeny of the selected F_2_ plants from the six populations will undergo further field evaluation and single-seed descent inbreeding to recover highly resistant genotypes.

## Materials and methods

### First greenhouse trial: F_1_ diallel

Eleven HRW wheat parents, which included 10 elite HRW wheat inbred lines from the NDSU breeding program and the cultivar, ND Noreen, was used following the appropriate institutional guidelines. They were tested for the likely presence of the known FHB resistance QTL, *Fhb1* and *Qfhs.ifa-5A,* in DNA marker screens performed by the North Central Small Grains Genotyping Laboratory (USDA ARS Cereal Crops Research Unit, Fargo, ND). Parent 19Nord-131 tested positive for both genes and 20Jun-P17 tested positive for *Fhb1*. The remaining nine lines tested negative for both genes. The eleven parents were crossed in all possible (55) non-reciprocal cross combinations. The 11 parents and 55 F_1_ were planted (5-inch diameter pots) in a randomized block design with four replicates in September 2021, and vernalized for 60 days before being transferred to a greenhouse. Each replication (single pot) consisted of two F_1_ plants of a specific cross combination. The plastic pots were filled with a soil mix comprising sphagnum peat moss (85%), perlite, vermiculite, limestone, and a wetting agent, and were wetted with a standard nutrient solution (Miracle-Gro® Professional Peat-Lite® Special 20–10-20), prepared according to the manufacturer's instructions (The Scotts Company, 1411 Scottslawn Road, Marysville, OH 43041). Slow-release fertilizer (Multicote-6 14-14-16, Haifa Chemicals Ltd, Israel) was added to the pots. FHB inoculation (four spikes per plant, including the main tiller) and the subsequent evaluation of disease symptoms were done during the winter of 2022.

### Second greenhouse trial: F_2_ evaluation of chosen cross combinations

A second FHB greenhouse trial was done in the winter of 2023. The F_2_ offspring resulting from six cross combinations from the F_1_ trial were included, alongside their parents and two controls (from the F_1_ trial). The evaluation was done in two parts. First, the four parents and two controls were planted in a completely randomized block design with four replicates. Each replicate consisted of a single pot containing three plants, resulting in 12 plants per entry. Second, the six F_2_ families were evaluated in six separate blocks, each comprising 22 pots. Each pot contained three F_2_, resulting in 66 plants per cross. All seeds were planted in September 2022 and vernalized for 60 days before being transferred to a greenhouse. The FHB inoculation was carried out in the winter of 2023, using four spikes per plant (including the main tiller).

### FHB inoculation

The single spikelet injection method described by Chu et al.^23^ was employed to inoculate wheat spikes at anthesis. A spore mixture (roughly equal quantities) of four *Fusarium graminearum* isolates (*Fg_124_1*, *Fg10_135_5*, *Fg13_79*, and *Fg08_13*) was prepared and provided by the Department of Plant Pathology at NDSU. A syringe was used to inoculate 10 μl of the *F. graminearum* macro-conidial spore suspension (± 100,000 conidia per ml) at anthesis into four spikes per plant. Inoculated spikes were covered with small plastic bags to maintain high humidity for three days. To indicate the date of inoculation, a small portion of the awn tips was cut off and a self-adhesive label was wrapped around the tiller. Twenty-one days after inoculation, the percentage of spikelets with visually discernible disease symptoms (DS = FHB disease severity) was calculated for each treated spike by dividing the number of infected spikelets by the total number of spikelets and multiplying the result by 100.

### Statistical analyses

Statistical analyses were done using the R computing software (Version 4.2.4^36^). An analysis of variance and combining ability was performed using Griffing’s design II (parents and one set of crosses (non-reciprocal)) assuming fixed effects^28^. Baker’s^33^ ratio of variances, 2MS_GCA_/(2MS_GCA_ + MS_SCA_) was calculated. This ratio was based on expected values of mean squares under a fixed model and was utilized to evaluate the relative significance of additive and nonadditive genetic effects. The data were also analyzed following the Hayman^29^ method which estimates the components of genetic variation (Table 6).

### Supplementary Information

Supplementary Information 1.

Supplementary Information 2.

## Data Availability

Data will be made available on request by contacting the corresponding author at bipin.neupane@wsu.edu.

## References

[1] Statistica. Global wheat production https://www.statista.com/statistics/267268/production-of-wheat-worldwide-since-1990/ (2023).

[2] Faris JD, Zhang Q, Chao S, Zhang Z, Xu SS (2014). Analysis of agronomic and domestication traits in a durum × cultivated emmer wheat population using a high-density single nucleotide polymorphism-based linkage map. Theor. Appl. Genet..

[3] Eat Wheat. Wheat foods around the world. https://eatwheat.org/learn/wheat-foods-around-the-world/ (2023).

[4] Halecki W, Bedla D (2022). Global wheat production and threats to supply chains in a volatile climate change and energy crisis. Resources.

[5] Foley JA (2011). Solutions for a cultivated planet. Nature.

[6] Luo K (2023). Molecular advances in breeding for durable resistance against pests and diseases in wheat: Opportunities and Challenges. Agronomy.

[7] Alberta. Fusarium head blight—Overview. https://www.alberta.ca/fusarium-head-blight-overview.aspx (2023).

[8] Ma Z (2020). Germplasms, genetics, and genomics for better control of disastrous wheat fusarium head blight. Theor. Appl. Genet..

[9] McMullen M, Jones R, Gallenberg D (1997). Scab of wheat and barley: A re-emerging disease of devastating impact. Plant Dis..

[10] McMullen M (2012). A unified effort to fight an enemy of wheat and barley: Fusarium head blight. Plant Dis..

[11] McCormick SP, Leonard KJ, Bushnell WR (2003). The role of DON in pathogenicity. Fusarium Head Blight of Wheat and Barley.

[12] Shah L (2018). Integrated control of fusarium head blight and deoxynivalenol mycotoxin in wheat. Plant Pathol..

[13] Stack RW, Leonard KJ, Bushnell WR (2003). History of fusarium head blight with emphasis on North America. Fusarium Head Blight of Wheat and Barley.

[14] Schroeder HW, Christensen JJ (1963). Factors affecting resistance of wheat to scab caused by *Gibberella zeae*. Phytopathology.

[15] Mesterhazy A (1995). Types and components of resistance to fusarium head blight of wheat. Plant Breed..

[16] Bai G, Shaner G (2004). Management and resistance in wheat and barley to fusarium head blight 1. Annu. Rev. Phytopathol..

[17] Paudel B (2020). WFhb1-1 plays an important role in resistance against fusarium head blight in wheat. Sci. Rep..

[18] Cuthbert PA, Somers DJ, Brulé-Babel A (2007). Mapping of Fhb2 on chromosome 6BS: A gene controlling fusarium head blight field resistance in bread wheat (*Triticum aestivum* L.). Theor. Appl. Genet..

[19] Su Z (2019). A deletion mutation in TaHRC confers *Fhb1* resistance to fusarium head blight in wheat. Nat. Genet..

[20] Waldron BL, Moreno-Sevilla B, Anderson JA, Stack RW, Frohberg RC (1999). RFLP mapping of QTL for fusarium head blight resistance in wheat. Crop Sci..

[21] Buerstmayr H (2003). Molecular mapping of QTLs for fusarium head blight resistance in spring wheat. II. Resistance to fungal penetration and spread. Theor. Appl. Genet..

[22] Schweiger W (2013). Transcriptomic characterization of two major Fusarium resistance quantitative trait loci (QTLs), *Fhb1* and *Qfhs.ifa-5A*, identifies novel candidate genes. Mol. Plant Pathol..

[23] Chu C (2011). Identification and molecular mapping of two QTLs with major effects for resistance to fusarium head blight in wheat. Theor. Appl. Genet..

[24] Ganaparthi, V. R., Adhikari, S., Marais, F., Neupane, B. & Bisek, B. The use of PI 277012-derived Fusarium head blight resistance QTL in winter wheat breeding. *Heliyon*, **9**(4) (2023). 10.1016/j.heliyon.2023.e15103 PMC10119711 37089302

[25] Eckard JT (2015). Native fusarium head blight resistance from winter wheat cultivars ‘Lyman’, ‘Overland, ‘Ernie’, and ‘Freedom’ mapped and pyramided onto ‘Wesley’-Fhb1 backgrounds. Mol. Breed..

[26] Townsend T (2013). The use of combining ability analysis to identify elite parents for *Artemisia annua* F_1_ hybrid production. Plos One..

[27] Suvi WT, Shimelis H, Laing M, Mathew I, Shayanowako AI (2020). Determining the combining ability and gene action for rice yellow mottle virus disease resistance and agronomic traits in rice (*Oryza sativa* L.). Agronomy.

[28] Griffing B (1956). Concept of general and specific combining ability in relation to diallel crossing systems. Aus. J. Biol..

[29] Hayman BI (1954). The analysis of variance of diallel tables. Biometrics.

[30] Jinks JL (1954). The analysis of heritable variation in a diallel cross in *Nicotiana rustica* varieties. Genet..

[31] Turney, S. Central limit theorem | formula, definition & examples. https://www.scribbr.com/statistics/central-limit-theorem/ (2022).

[32] Zheng N (2022). Analysis of *Fhb1* gene and resistance to fusarium head blight in 3,177 diverse wheat accessions. J. Cereal Sci..

[33] Baker RJ (1978). Issues in diallel analysis. Crop Sci..

[34] Atnaf M, Tesfaye K, Dagne K, Mohammed H (2014). Hyman’s diallel analysis to study genetic parameters of phenological traits in common bean (*Phaseolus vulgaris*). Int. J. Agric. Sci. Nat. Res..

[35] Graf RJ (2013). Emerson hard red winter wheat. Can. J. Plant Sci..

[36] R Core Team. R: A language and environment for statistical computing. R foundation for statistical computing, Vienna, Austria. https://www.R-project.org/ (2022).

