# Supplementary material for: A diallel study to detect genetic background variation for FHB resistance in winter wheat
Source: Sci Rep. 2024 Feb 26;14:4614. doi: 10.1038/s41598-024-53710-z (PMC10897133; doi:10.1038/s41598-024-53710-z)
Supplement: Supplementary file 1 — Supplementary Information 1. [file 41598_2024_53710_MOESM1_ESM.pdf]

Phil L. Bruckner, Professor
Department of Plant Sciences & Plant Pathology
Montana State University
Bozeman, MT 59715-3140
bruckner@montana.edu
PHONE 406-994-5127, FAX 406-994-1848

MEMORANDUM

TO: Wheat Cultivar Release & Recommendation Committee

FROM: Phil Bruckner and Jim Berg, Winter wheat breeders

DATE: December 28, 2010

RE: Proposal for recommendation of Decade hard red winter wheat

The following motion and supporting documentation is presented for consideration at the 2011 MAES Cultivar Release and Recommendation Meeting in Bozeman:

Motion: That Decade hard red winter wheat be recommended for production in Montana cropping districts 3, 4, 5, and 6.

‘Decade’ Winter Wheat

Phil Bruckner and Jim Berg, Winter Wheat Breeding Program, Montana State University

Updated 12/2010

Decade is a white-chaffed hard red winter wheat developed by the Montana Agricultural Experiment Station and released to seed growers in 2010. Decade is a joint release with the North Dakota Agricultural Experiment Station. Decade was selected from a composite of three closely related F1 populations. Decade is a very high yielding (Table 1) winter hardy variety well adapted to western North Dakota and eastern Montana with medium to high test weight, early maturity, reduced height, and medium to high grain protein (Table 2). Decade has excellent milling and baking quality (Table 3). It is resistant to stem rust, but susceptible to stripe rust. Relative to CDC Falcon, Decade is equivalent in yield potential and winter survival, with improved test weight, earlier maturity, higher grain protein content, superior milling characteristics and higher water absorption. PVP, Title V is pending.

Table 1. Yield of Decade, 2007-2010, compared to a set of recommended winter wheat varieties.

Variety	Districts							All Locations
	**1** Kalispell	**2** Bozeman[1/]	**3** Huntley[2/]	**4** Moccasin[3/]	**5** Conrad[4/]	**5** Havre[5/]	**6**- Sidney & Williston	
location-years	4	7	20	13	6	9	6	65
**Yellowstone**	**126.3****	**68.6****	**74.6***	**54.6****	**68.7****	**60.2***	**50.7***	**69.2****
**Wahoo**	113.8	**64.2***	**75.5****	50.2	**66.6***	**62.8****	49.3	**67.4***
**Decade**	112.5	61.4	**75.0***	**52.8***	**66.7***	**60.2***	**52.2***	**67.3***
**Jagalene**	**124.2***	57.9	**73.7***	51.6	61.9	**59.3***	42.8	65.5
**CDC Falcon**	**118.4***	59.5	68.9	48.9	**66.0***	58.6	**54.0***	64.6
**Pryor**	109.0	**63.4***	70.9	51.3	**67.2***	56.3	44.8	64.5
**Promontory**	**120.6***	**63.9***	69.3	50.4	58.9	57.2	42.6	63.8
**Norris (CL)**	109.1	58.2	**73.1***	48.2	62.1	55.0	46.0	63.5
**Ledger**	116.4	60.3	70.7	46.9	61.9	57.2	40.3	62.8
**Neeley**	106.5	60.5	69.0	47.3	62.4	52.4	45.0	61.6
**Jerry[6/]**	99.2	57.8		47.3	59.6	53.8	**55.5****	
**LSD (0.05)**	**12.1**	**5.6**	**4.3**	**3.2**	**5.4**	**3.9**	**5.8**	**2.0**

** = indicates highest value within a column

* = indicates varieties with values equal to highest variety within a column based on Fisher's protected LSD (p=0.05)

1/ includes data from Dry Creek, Willow Creek

2/ includes data from Forsyth, Hardin area, Lodge Grass, Molt, Rapelje

3/ includes data from Denton, Geraldine, Winifred

4/ includes data from The Knees, Shelby

5/ includes data from North Havre, Loma

6/ Jerry not grown at Huntley (SARC) in 2008

Table 2. Agronomic characteristics of Decade, 2007-2010, compared to a set of recommended winter wheat varieties

Variety	Test weight lb/bu	Winter survival %	Heading date Julian	Heading date Calendar	Plant height in	Lodging score 1/ (0-9)	Protein %	Sawfly cutting %	Stripe rust %	Coleoptile length in
location-years	64	4	34		63	6	66	4	5	3
**CDC Falcon**	59.7	**50***	165.9	15-June	30.1	1.7	12.1	21	9	2.8
**Decade**	60.6	**53***	164.6	14-June	31.8	2.2	**12.4****	18	16	3.0
**Jagalene**	**61.8****	30	164.1	13-June	31.7	2.8	12.2	23	7	3.1
**Jerry**[2/]		**57****						34	11	3.1
**Ledger**	60.6	29	165.9	15-June	31.3	4.0	11.8	23	12	3.2
**Neeley**	59.8	44	168.2	17-June	35.3	4.8	11.9	23	25	3.4
**Norris (CL)**	61.2	36	163.3	12-June	34.9	3.3	12.1	23	18	3.4
**Promontory**	**61.6***	30	166.0	15-June	33.4	3.2	11.5	39	1	2.6
**Pryor**	59.7	40	167.7	17-June	30.6	1.9	11.6	9	24	2.8
**Wahoo**	59.1	48	162.7	12-June	32.3	3.1	11.8	27	11	2.8
**Yellowstone**	59.8	45	167.5	16-June	33.7	3.0	11.9	22	1	2.6
**LSD (0.05)**	**0.3**	**9**	**0.5**		**0.5**	**1.7**	**0.2**	**13**	**16**	**0.2**

** = indicates highest value within a column

[1/] 0 = best

* = indicates varieties with values equal to highest variety within a column based on Fisher's protected LSD (p=0.05)

[2/] Jerry not grown at Huntley in 2008

Table 3. Mill and bake characteristics of Decade, 2007-2009, compared to a set of recommended winter wheat varieties

Variety	PPO [1/]	Kernel hardness	Flour yield %	Flour protein %	Flour Ash %	Mixograph mix time min	Mixograph absorption %	Baking mix time min	Baking absorption %	Loaf volume cc
location-years	12	12	12	12	12	12	12	12	12	12
**CDC Falcon**	0.991	66.8	66.1	11.2	0.43	4.9	61.2	8.8	71.2	**1083***
**Decade**	0.980	73.3	68.7	**11.8****	**0.40****	7.6	**65.9****	16.3	**76.4****	**1095****
**Jagalene**	0.999	75.5	70.9	11.5	0.41	4.5	62.2	6.2	72.3	**1066***
**Ledger**	0.870	69.7	**72.0****	11.0	**0.40****	5.0	61.4	9.3	71.5	**1066***
**Neeley**	0.746	73.5	66.7	11.2	0.42	4.9	61.3	6.8	71.4	1036
**Norris (CL)**	0.794	72.7	68.8	11.2	**0.40***	4.9	61.6	7.7	72.3	1025
**Promontory**	0.280	76.5	70.4	10.7	0.41	4.9	60.8	6.3	70.6	1039
**Pryor**	0.876	75.4	69.1	10.8	**0.40***	3.3	60.1	4.5	69.0	1018
**Wahoo**	0.892	71.8	67.8	11.1	0.41	4.6	61.2	6.3	70.9	994
**Yellowstone**	0.684	78.2	69.0	11.1	0.41	8.3	62.8	13.0	73.7	**1088***
**LSD (0.05)**	**0.130**	**2.8**	**0.8**	**0.3**	**0.01**	**0.8**	**1.0**	**1.6**	**1.2**	**32**

** = indicates highest value within a column

* = indicates varieties with values equal to highest variety within a column based on Fisher's protected LSD (p=0.05)

[1/] low is best for noodles
