# Supplementary material for: A diallel study to detect genetic background variation for FHB resistance in winter wheat
Source: Sci Rep. 2024 Feb 26;14:4614. doi: 10.1038/s41598-024-53710-z (PMC10897133; doi:10.1038/s41598-024-53710-z)
Supplement: Supplementary file 2 — Supplementary Information 2. [file 41598_2024_53710_MOESM2_ESM.pdf]

FACT SHEET

ARMOUR WHEAT

Armour is a new hard red winter variety developed by WestBred® and licensed to AGSECO. Armour is broadly adapted to the central Great Plains and features early maturity and excellent yield potential. This variety offers exceptional straw strength, very good leaf rust resistance, and excellent tolerance to low pH soil conditions. Armour is an "Impact" variety and should grow in popularity to dominate acres planted in eastern Colorado, all of Kansas, the northern 1/2 of Oklahoma, and southern Nebraska.

Characteristics

Plant Height:	Medium
Maturity:	Early
Coleoptile Length:	Medium
Seed Size:	Large
Straw Strength:	Excellent
Test Weight:	Good
Winterhardiness:	Very Good
Milling & Baking:	Acceptable
Yield Potential:	Excellent
Grazing Potential:	Good

Disease & Insects

Barley Yellow Dwarf:	Intermediate
Hessian Fly:	Susceptible
Powdery Mildew:	Mod. Resistant
Leaf Rust:	Resistant
Septoria Leaf Blotch:	Mod. Susceptible
Spindle Streak Mosaic:	Resistant
Soil Borne Mosaic:	Resistant
Stripe Rust:	Resistant
Tan Spot:	Mod. Resistant
Wheat Streak Mosaic:	Mod. Susceptible

Pedigree: WestBred Exp. Line, KS94U326.

PVPA Status: Protected by patent, must be sold only as a class of certified seed.

WestBred® is a registered trademark of Monsanto Company
